# Cluster based prediction of PDZ-peptide interactions

**DOI:** 10.1186/1471-2164-15-S1-S5

**Published:** 2014-01-24

**Authors:** Kousik Kundu, Rolf Backofen

**Affiliations:** Bioinformatics Group, Department of Computer Science, University of Freiburg, Kragujevac, Germany; Centre for Biological Signalling Studies (BIOSS), University of Freiburg, Kragujevac, Germany; Centre for Biological Systems Analysis (ZBSA), University of Freiburg, Kragujevac, Germany; Center for non-coding RNA in Technology and Health, University of Copenhagen, Grønnegårdsvej 3, DK 1870 Frederiksberg C, Denmark

**Keywords:** PDZ domain-peptide interactions, protein recognition modules, protein domain clustering, semi-supervised learning, support vector machines

## Abstract

**Background:**

PDZ domains are one of the most promiscuous protein recognition modules that bind with short linear peptides and play an important role in cellular signaling. Recently, few high-throughput techniques (e.g. protein microarray screen, phage display) have been applied to determine in-vitro binding specificity of PDZ domains. Currently, many computational methods are available to predict PDZ-peptide interactions but they often provide domain specific models and/or have a limited domain coverage.

**Results:**

Here, we composed the largest set of PDZ domains derived from human, mouse, fly and worm proteomes and defined binding models for PDZ domain families to improve the domain coverage and prediction specificity. For that purpose, we first identified a novel set of 138 PDZ families, comprising of 548 PDZ domains from aforementioned organisms, based on efficient clustering according to their sequence identity. For 43 PDZ families, covering 226 PDZ domains with available interaction data, we built specialized models using a support vector machine approach. The advantage of family-wise models is that they can also be used to determine the binding specificity of a newly characterized PDZ domain with sufficient sequence identity to the known families. Since most current experimental approaches provide only positive data, we have to cope with the class imbalance problem. Thus, to enrich the negative class, we introduced a powerful semi-supervised technique to generate high confidence non-interaction data. We report competitive predictive performance with respect to state-of-the-art approaches.

**Conclusions:**

Our approach has several contributions. First, we show that domain coverage can be increased by applying accurate clustering technique. Second, we developed an approach based on a semi-supervised strategy to get high confidence negative data. Third, we allowed high order correlations between the amino acid positions in the binding peptides. Fourth, our method is general enough and will easily be applicable to other peptide recognition modules such as SH2 domains and finally, we performed a genome-wide prediction for 101 human and 102 mouse PDZ domains and uncovered novel interactions with biological relevance. We make all the predictive models and genome-wide predictions freely available to the scientific community.

**Electronic supplementary material:**

The online version of this article (doi:10.1186/1471-2164-15-S1-S5) contains supplementary material, which is available to authorized users.

## Background

Protein-protein interactions are the most essential cellular process in eukaryotes that involve many important biological activities such as signal transduction, maintaining cell polarity etc [1–3]. Many protein-protein interactions in cellular signaling are mediated by modular protein domains. Peptide recognition modules (PRMs) are an important subclass of modular protein domains that specifically recognize short linear peptides to mediate various post translation modifications [4].

PDZ domains are one of the most widespread peptide recognition modules (PRMs) that predominantly found in signaling proteins in multi-cellular organisms and play an important role in the establishment of cell polarity, neuronal signaling, protein trafficking etc [1–3]. It has also been reported previously that PDZ domains take a pivotal role in several human diseases such as schizophrenia, cystic fibrosis etc [5]. The name PDZ was derived from the acronym of three proteins, namely postsynaptic density protein-95 (PSD-95), disks large tumor suppressor (DLG1) and zonula occludens-1(ZO-1) [6–8]. PDZ domains are typically 80-90 amino acids in length, containing 5-6 *β* strands and 2 *α* helices. The second *β* strand, second *α* helix and a GLGF loop of the PDZ domains collectively form the binding pocket, which recognizes the hydrophobic C-terminal peptide of a target protein [9, 10]. Albeit many PDZ domains preferentially recognize the C-terminal tails of their target proteins, other interaction patterns have also been described [3, 11]. In earlier studies, PDZ domains were grouped into different specificity classes based on their target motif structures: *X[T/S]Xφ-COOH* (Class I motif), *XφXφ-COOH* (Class II motif) and a minor *X[D/E]Xφ-COOH* (Class III motif), where *X* represents any natural amino acid and *φ* represents hydrophobic amino acid [9, 12]. Nevertheless, this classification system is an oversimplification since it is known that every residue in the target peptide contributes to the binding specificity [13, 14].

To determine binding specificity of PDZ-peptide interactions, several high-throughput techniques (e.g. protein microarray screening, phage display etc.) have been employed [15, 16]. While the enormous amount of data generated by these high-throughput experiments have become invaluable to build powerful computational models for predicting domain-peptide interactions, these kinds of data also have some severe caveats. First, data maybe rich only for certain domains while it is scarce or completely missing for others. Second, reliable information is usually gained only for positive interactions, while there is lack of negative information, leading to a class imbalance problem. Thus, it is still an open problem to build general predictive models that have high specificity for PDZ-peptide interactions using this kind of data.

We can distinguish two types of approaches to tackle this problem. The first type uses structural information to improve specificity. It was recently observed that structural information can be used to improve the prediction of binding sites for DNA-binding or RNA-binding proteins (e.g. [17] for DNA-binding proteins, and [18, 19] for RNA-binding proteins). More specifically for the PDZ domain, *Chen et al*. used structural information from a reference PDZ-peptide complex structure to build a Bayesian model for predicting PDZ-peptide interactions [20]. Recently, Bader and coworkers developed several support vector machine based approaches to predict PDZ-peptide interactions [21–23]. These methods are based on only one reference PDZ-peptide complex structure and thus do not perform well for all PDZ domains. Other structure-based approaches are computational very expensive and depend on solved structures, which in reality are very few [24, 25].

The other type consists of approaches that cope with the problem of few data points by combining the experimental evidence for domains with similar binding preference. As was recently shown by two high-throughput experiments, PDZ domains can be classified into 16 different specificity classes [15, 16] with similar ligand binding profiles. *Stiffler et al*. developed a multi-domain selectivity model (MDSM) in 2007, which uses in effect a mixture of position specific scoring matrices (PSSMs) [15]. However, PSSM based models have several drawbacks as they are essentially linear models and thus unable to consider the positional correlation between the amino acid positions in the binding peptides. Furthermore, the models are based only on the positive interaction data and hence do not use of information from negative interaction data. Recently, few methods have been developed to overcome this problem by using a support vector machine [26, 27]. *Li et al*. applied a nearest neighbor approach (based on domain sequence identity and ligand binding specificity) to extend the training set for each domain, building models for 174 PDZ domains in total [27].

In this work, we present a cluster based prediction of PDZ-peptide interactions for human (*H. sapiens*), mouse (*M. masculas*), fly (*D. melanogaster*) and worm (*C. elegans*), using a machine learning approach. The importance of our method is five fold: (i) clustering of a very large set of PDZ domains based on their sequence identity. This comprehensive study allowed us to construct specialized models for 43 PDZ families, covering 226 PDZ domains, which are more accurate compared to the state-of-the-art and offers models for the largest set of PDZ domains to date. (ii) The data obtained from high-throughput experiments are often found to lack of non-interacting data (i.e. negative data) and thus lead to a great class imbalance problem. Previous research showed that the performance of many machine learning methods are significantly poorer on highly imbalanced data [28–30]. To deal with this issue we employed a semi-supervised machine learning approach to identify high confidence negative interactions. (iii) We allowed the dependency between the amino acid positions in the binding ligand. (iv) We built two types of models, one sequence-based and one based on contact information from reference structures, and compared the performance of these models. Surprisingly, no significant difference in predictive performance was observed. (v) Finally, we performed a genome-wide analysis for 101 and 102 PDZ domains from human and mouse, respectively and uncovered novel, biological meaningful, PDZ-peptide interactions.

## Methods

### PDZ domain data

For retrieving all the annotated PDZ domains from human, mouse, fly and worm proteomes, we used UniProtKB/Swiss-Prot database, which is a well known manually curated and reviewed database [31]. At the time of analysis, the UniProtKB/Swiss-Prot database, release 2013-01, contained 20248 human (*H. sapiens*), 16597 mouse (*M. masculas*), 3182 fly (*D. melanogaster*) and 3382 worm (*C. elegans*) proteins. A large set of 548 PDZ domains, comprising 271 human, 234 mouse, 27 fly and 16 worm PDZ domains, were derived.

### Clustering of PDZ domains

We clustered all the available PDZ domains using Markov clustering algorithm (MCL) based on their global sequence identity [32]. MCL is a popular and efficient method for clustering biological sequences and was successfully applied for clustering of protein families [33]. More recently, *Li et al*. have proposed that PDZ domain pairs with greater than 50% sequence identity share similar binding specificity [27]. Thus, we defined 50% sequence identity as a cut-off value to represent similar specificity. We used Needleman-Wunsch algorithm in order to calculate pairwise sequence identity of all PDZ domains. PDZ domain pairs with less than 50% sequence identity were discarded to reduce noise [34]. In the MCL method, PDZ domain sequence identities can be considered as a weighted graph, where the domains are the nodes and the identity relationships are the edges. Since the MCL algorithm specifically designed for the simple and weighted graphs, clustering of the PDZ domains using MCL is highly reliable. We applied MCL algorithm with 1.4 inflation parameter. This parameter was used for controlling the granularity or the tightness of the clusters and we found 1.4 as the best inflation value for clustering of PDZ domains. Only families with at least two PDZ domain sequences were considered. Finally, 515 PDZ domains from human, mouse, fly and worm were classified into 138 different families.

### Domain-Peptide interaction data

#### Dataset I

We used a protein microarray screening data to analyze the specificity of PDZ domains, comprising 157 mouse PDZ domains and 217 fluorescently labeled genome-encoded peptides [15]. The initial interaction data derived from microarray screening was further analyzed by fluorescence polarization. Apparent equilibrium dissociation constant (K_*D*_ value) was applied to determine the positive and negative classes [15]. A total 731 positive interactions and 1361 negative interactions were derived that involved 85 mouse PDZ domains and 181 peptides, using a K_*D*_ cutoff of 100 *µ*M. We used same K_*D*_ cutoff value as mentioned in [15].

#### Dataset II

For the human phage display experiment, we considered a total of 1389 interactions that involved 54 human PDZ domains and 1211 peptides [16]. Note that this experiment provides only positive interaction data. Thus we did not have any negative interaction data for this dataset.

#### Dataset III

From PDZBase [35], which is a high quality known PDZ-peptide interaction database, we extracted non-redundant 201 interactions, which were composed of 94 domains and 115 peptides. We considered interaction data only from human, mouse, fly and worm. Note that PDZBase also contains only positive interaction data and hence no negative interaction data was available in this database as well.

### Domain-peptide complex structures

For retrieving all the available PDZ-peptide complex structures, we used Protein Data Bank (PDB), which contains experimentally solved protein structures [36]. At the moment of analysis PDB contained 55 PDZ-protein and/or PDZ-peptide complex structures comprising 47, 5 and 3 structures from human, mouse and fly, respectively. Note that we were unable to find any PDZ-peptide complex structure for worm. After filtering according to available interaction data, we were left with 21 human, 5 mouse and 3 fly PDZ-peptide complex structures.

### Dataset compilation

We have combined all the positive and negative interaction data from dataset I, dataset II and dataset III. Five C-terminal residues of the peptides were considered since they are the most important for determining PDZ domain specificity [9, 16]. Finally, we retrieved a total 3592 interactions involved 194 domains and 1437 peptides.

### Semi-supervised negative data

Datasets derived from high-throughput experiments usually suffer from a lack of reliable negative interaction data. In our study, we were only able to obtain the negative interaction data from a microarray experiment although the dataset had an imbalanced problem. Other data sources (i.e. phage display and PDZBase) provide only positive interaction data. Previous study showed that machine learning methods work poorly when the dataset is highly imbalanced [28–30]. In order to generate more negative data we have employed a semi-supervised learning approach (SSL) that was also implemented successfully in our previous work [30]. The general strategy of SSL is to learn from a small amount of labeled data and a large amount of unlabeled data. Here, differently from the general problem formulation for SSL, we were interested in using the unsupervised material to have a better characterization only of the minority class; in our case, the negative class. Albeit, there are several strategy to deal with SSL problem, we have chosen the self-training approach that relies only on the good discriminative properties of the base classifier and thus fits well with our datasets. The method is a simple wrapper scheme around a base classifier: the initial labeled data is used to train the classifier which then assigns a label to the unlabeled material. Since dataset I was only comprising of mouse PDZ-peptide interaction data, we used all the C-terminal peptides from mouse proteome as unlabeled data.

Finally, the predicted unlabeled peptides having the probability of 0.5 to 0.8 towards the negative class were considered. We ignored very high scoring (probability more than 0.8) predicted negative peptides since they might be very far from positive class and therefore could produce low quality models. We randomly chose negative data from the pool of predictive negatives, added them to the training data, and re-trained the classifier. In general, there was five times more negative data than positive data [37].

Note that we need both positively and negatively labeled data to apply the described SSL approach since we need to train the base classifier with both positive and negative data. Hence, we could only employ the SSL approach to domains that occur in dataset I as it contains both classes. For those PDZ domains where only the positive data was available, we chose the negative data randomly from C-terminal peptides of the respective organism from UniProtKB/Swiss-Prot [31]. Note that we only used the negative interaction data from the semi-supervised learning for the training sets, while our test sets contained only experimentally verified positive and negative interaction data.

### Feature encoding

Previous studies show that the C-terminal residues of a peptide are the most important for PDZ-peptide binding specificity [9]. We followed the literature and restricted the peptide sequence to 5 C-terminal positions, namely we extracted the amino acids in positions from P0 to P-4 downstream, where the P0 is the extreme last C-terminal position. We have developed two types of feature encoding methods: i) sequence-based and ii) contact-based feature encoding.

In the sequence-based feature encoding, a peptide sequence was mapped into a binary vector *x*, living in a 20 × 5 = 100 dimensional space. I.e., for each position, we reserved 20 dimensions (one for each amino acid type) and encoded the amino acid type with a 1 in the corresponding dimension and 0 elsewhere.

For the contact-based feature encoding, we used an approach similar to the one described by *Chen et al*. [20]. Here, the important position pairs (one amino acid from the domain and another from the peptide) were taken into account. First, we constructed a cluster-based, PDZ domain, multiple sequence alignments using *MAFFT* [38]. We then considered the core position pairs that are in close proximity and hence extracted only the position pairs with distance less than 4.5 angstroms using domain-peptide complex structures. Note that we have used different reference structures for different families. Each position pair was encoded as a binary vector *x*, living in a 20 × 20 = 400 dimensional space. All the position pairs were then encoded in a binary vector of size 400 × *n*, where *n* is the number of binding pairs. Finally the sequence-based encoding was concatenated with the contact-based encoding, which produced a binary vector of size 100 + 400 × *n*.

For each domain *D*, we have compiled a data set encoded as a set of pairs (*x*_1_,*c*_1_),..,(*x*_*n*_*,c*_*n*_) where, *x*_*i*_ is the binary feature vector for peptide *P*_*i*_ with the class label *c*_*i*_ ∈ {*−*1, 1}. The class label is +1 if the domain *D* interacts with peptide *P*_*i*_ and -1 otherwise.

### Performance measures

We formulated a learning problem for each PDZ domain family. The predictive performance for each problem was assessed by computing 5 measures: sensitivity, specificity, precision, area under the receiver operating characteristics curve and area under the precision recall curve. These are defined as: , , , where TP denotes true positive, FP denotes false positive, TN denotes true negative and FN denotes false negative. The area under the receiver operating characteristics curve (AUC ROC) is defined as the area under the curve obtained by plotting the fraction of true positives out of the positives (TPR = true positive rate) vs. the fraction of false positives out of the negatives (FPR = false positive rate), at various threshold settings. The area under the precision recall curve (AUC PR) is defined as the area under the curve obtained by plotting precision as a function of recall.

## Results and discussion

### Tree of PDZ domains

In recent years, enormous amounts of interaction data have been generated by various high-throughput experiments thus computational methods are invaluable to analyze these data. One of the major problems while analyzing these data is sufficient amounts of data may be available for certain domains but completely missing or much less available for another domain. For example, there are only two positive interactions for PDZ 1 and PDZ 2 domains of human DLG2 and DLG4 available in the literature. To overcome this limitation our first goal was to combine the PDZ domains that are similar in substrate specificity and therefore build a single classifier for these similar domains. Hence, this approach enables us to make separate models for each domain family.

**Figure 1:**
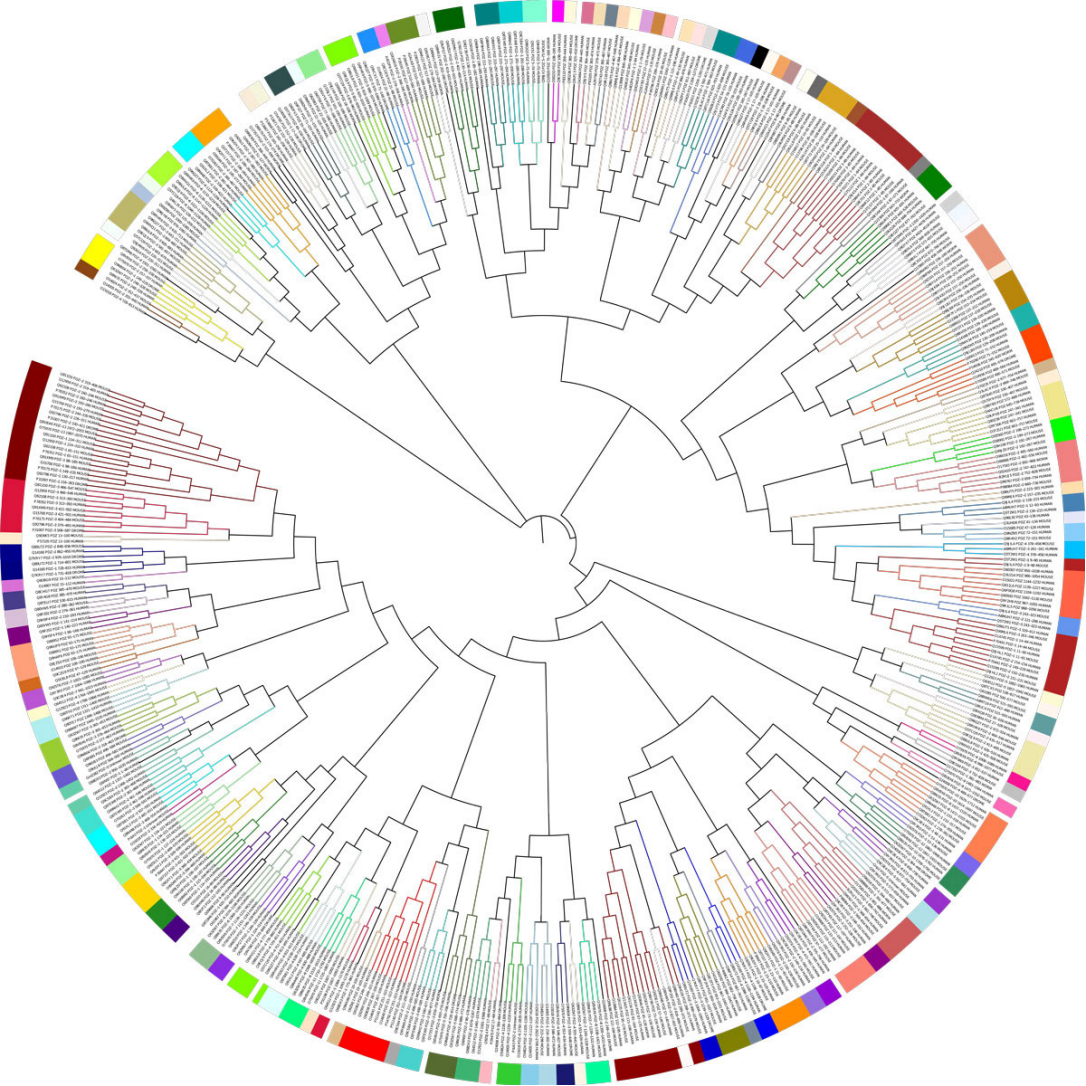
**Clustering of PDZ domains**. Phylogenetic tree of all available PDZ domains from human, mouse, fly and worm. The MCL clustering output was mapped onto the phylogenetic tree. A total number of 138 PDZ families are presented by 138 colors. iTOL was used for the visualization [45].

First, we aligned all available PDZ domains (human, mouse, fly and worm) annotated in UniProtKB/Swiss-Prot by using *MAFFT* and built a phylogenetic tree [38]. We then clustered all the similar PDZ domains based on their sequence identity by using Markov clustering algorithm (MCL) [32]. MCL is a fast and powerful algorithm for clustering biological sequences. 50% sequence identity was set for the cutoff value as previous research showed that the PDZ domains with more than 50% sequence identity have similar binding specificity [27] (see *Material and methods* for details). All the available PDZ domains (548) were classified into 138 families. Out of all 548 domains, we were unable to classify 33 PDZ domains since the sequences are too diverse. The biggest family consists of 20 PDZ domains from human, mouse and fly. Finally, we have mapped the 138 families on the phylogenetic tree of all PDZ domains for better visualization (see Figure 1). In this figure each family is represented by a different color. Additionally, we have described the peptide preferences for each PDZ domain family. Amino acid composition of the binding peptides was visualized using sequence logos [39], showing the amino acid enrichment at each position in the binding peptides. See Additional file 1: Figure S1 for the ligand binding specificity of each PDZ domain family.

### Modeling

We used two strategies for modeling our data, namely a purely sequence-based approach and a contact-based modeling that uses structural information.

### Sequence-based data modeling

For the sequence-based modeling approach, we followed the literature and considered five C-terminal residues of peptide sequences as an input, where the position of C-terminal residue is given P0 and going upstream P-1, P-2 and so on. To define the positional features, we extracted amino acids from peptides and mapped them into a binary vector *x* living in a 20 × 5 = 100 dimensional space (see *Materials and methods for details*). Families with at least 10 positive interaction data were considered for modeling. In summary, we built models for 43 families covering 226 PDZ domains.

### Contact-based data modeling

**Figure 2:**
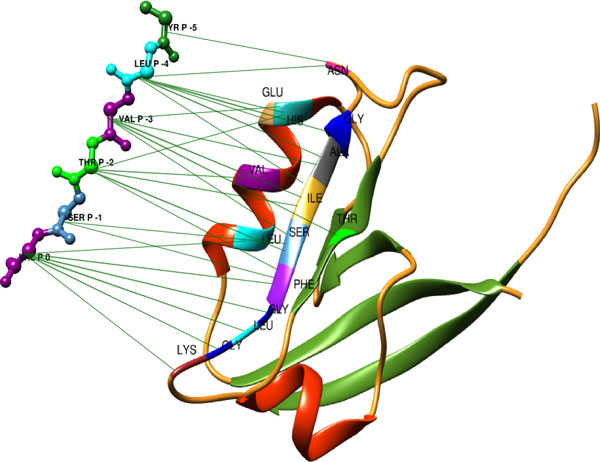
**PDZ-peptide complex structure**. Representative PDZ-peptide complex structure (PDB-id: 4G69) for PDZ family 1. 2nd PDZ domain from human DLG1 binds with C-terminal peptide of human APC protein. Green lines indicate the binding pairs with distance less than 4.5 angstroms. UCSF Chimera was used for the visualization [46].

The contact-based modeling approach combines the peptide sequence information with PDZ-peptide complex structure information. We followed the similar approach taken by *Chen et al*. in 2008 [20]. However, we did not use only one reference structure for all domains. Instead, we used a specific reference structure for each family by selecting one representative domain-peptide complex structure for each family from the PDB database [36]. For these domain-peptide structures, we considered only the position pairs (one amino acid from the domain and another from the peptide) with distance less than 4.5 angstroms (see Figure 2). The important position pairs were separately derived for each PDZ domain family. All position pairs were then encoded in a binary vector of size 400 × *n*, where *n* is the number of binding pairs (see *Materials and methods for details*). We concatenated sequence-based features with contact-based features and finally, we built models for 10 families covering 70 PDZ domains.

### Predictive model and performance evaluation

We employed a Gaussian kernel support vector machine to build predictive models [40]. SVM^*light*^ software was used to build the SVMs [41]. We used a 5-fold stratified cross-validation in order to evaluate the predictive performance of each model. Here, the data is partitioned into 5 parts ensuring the same proportional distribution of positive and negative instances in each part. Each part is then used in turn as a held out test set, while the remaining 4/5th of the data is used as training set. In the cross-validation step, only the families with at least 10 positive data and 10 negative data were taken into account so that each test set contains at least 2 positive and 2 negative interactions.

**Figure 3:**
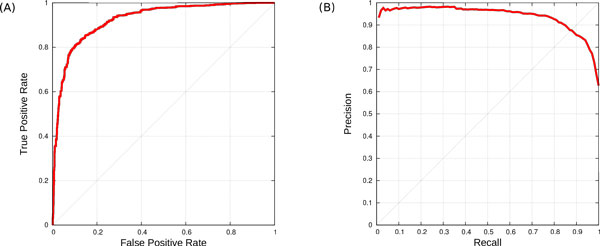
**Performance**. (A) The AUC-ROC and (B) the AUC-PR curve obtained by sequence-based feature encoding method.

For the sequence-based approach comprising models for 43 families covering 226 PDZ domains, only 22 families covering 136 PDZ domains met this criteria and therefore used in cross-validation. The hyper parameters (i.e. *γ* and the cost parameter *C*) for each fold were optimized using 5-fold grid search method over the training sets. See Additional file 1: Table S1 for the performances of all 22 families. We computed area under the ROC curve (AUC ROC) and area under the precision and recall curve (AUC PR) for the 22 families. Using sequence-based feature encoding, we achieved a very good average AUC ROC of 0.92 and AUC PR of 0.94 (see Figure 3).

For the contact-based feature encoding method comprising initially 10 families with 70 PDZ domains, only 6 PDZ families covering 39 PDZ domains met the selection criteria for the cross-validation. No significant differences were observed when we compared the performances (AUC ROC and AUC PR) of sequence-based and contact-based approaches (see Additional file 1: Table S1, S2 and Figure S2). Therefore, we can conclude that the peptide sequence information is sufficient to define the binding specificity of a PDZ domain on the current available data.

### Benchmarking of published methods

**Figure 4:**
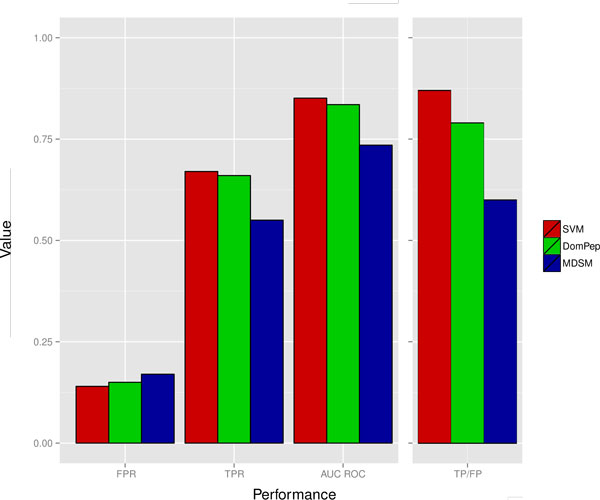
**Performance evaluation on an independent test set**. Performance comparison of tree different tools. Red, green and blue bars indicate the predicted performances by our tool (SVM), DomPep and MDSM, respectively. The figure clearly shows that our tool (SVM) achieved better performance.

We compared our results with two state-of-the-art tools, namely MDSM (multi-domain selectivity model) [15] and DomPep [27], on an independent test set. The independent test set contained 493 positive interactions and 3059 negative interactions that involved 74 mouse PDZ domains and 48 peptides [15]. Among them, we used interactions for 50 PDZ domains that were common in all three methods (MDSM, DomPep and our method). We make sure the peptides were not included in our training sets. Our models achieved a true positive rate (TPR) of 0.67, false positive rate (FPR) of 0.14 and AUC ROC of 0.85 with a true-positive/false-positive (TP/FP) ratio of 0.87 outperforming the other two approaches: MDSM achieved TPR of 0.55, FPR of 0.17 and AUC ROC of 0.74 with TP/FP ratio of 0.55; the DomPep achieved TPR of 0.66, FPR of 0.15 and AUC ROC of 0.84 with TP/FP ratio of 0.79 (see Figure 4).

In another experiment, we tested our method with MDSM on a validated dataset. We could not test DomPep since many of the test instances were present in the DomPep training set and hence a fair comparison was not possible. The test data was retrieved from an experimentally validated database, called PDZBase [35]. We compared 20 mouse PDZ-peptide interactions derived from PDZBase that were neither included in MDSM nor in our training set. Out of 20 interactions, we successfully predicted 14 interactions with a true positive rate (TPR) of 0.70, compared to only 4 interactions predicted by MDSM with a true positive rate (TPR) of 0.20. For calculating MDSM score, an unit threshold was defined as the ratio of the original prediction score (*φ*) over a scoring threshold (*τ*), specific for each domain [15]. A peptide was then predicted to bind with a PDZ domain *i*, if *φ*_*i*_*/τ*_*i*_*>*1. Table 1 lists the scores for all 20 validated interactions as calculated by MDSM and by our method.

**Table 1:** SVM and MDSM scores for validated set.

PDZ domain	Peptide	SVM score	MDSM score	Pubmed Ref.
Cipp-(3/10)	IESDV	**0.44**	-0.7	9647694
Cipp-(3/10)	LESEV	**0.30**	-0.62	9647694
Cipp-(3/10)	QQSNV	**0.29**	-0.78	9647694
Cipp-(3/10)	KEYYV	**0.51**	-0.34	9647694
Dvl1-(1/1)	SETSV	-1.27	-0.74	12490194
Pdlim5-(1/1)	DITSL	-0.24	-0.15	10359609
Erbin-(1/1)	LDVPV	**0.99**	0.61	10878805
Magi-2-(5/6)	KESSL	**1.76**	0.19	10681527
MUPP1-(10/13)	IATLV	**1.00**	0.46	11000240
MUPP1-(10/13)	GKDYV	**1.00**	**1.68**	11689568
NHERF-1-(1/2)	FDTPL	**1.06**	0.01	10980202
LIN-7A-(1/1)	IESDV	**0.33**	0.29	10341223
Lin7c-(1/1)	IESDV	**0.33**	1.00	10341223
ZO-3-(1/3)	GKDYV	**0.99**	0.09	10601346
a1-syntrophin-(1/1)	VLSSV	-1.47	0.16	11571312
PSD95-(1/3)	LQTEV	**0.38**	**1.41**	11937501
PSD95-(1/3)	NETVV	-1.35	**1.19**	12067714
PSD95-(1/3)	GETAV	-1.32	**1.23**	12067714
PSD95-(1/3)	EESSV	-2.23	0.77	11134026
PSD95-(1/3)	RTTPV	**1.00**	0.61	12359873

SVM and MDSM scores for experimentally validated interactions derived from PDZBase [35]. A peptide is predicted to bind to a PDZ domain if the score is more than 0 for SVM and more than 1 for MDSM. Bold numbers indicate true positive interactions.

The advantages of our approach compared to aforementioned tools are threefold: i) using an accurate clustering approach allows our method to achieve a higher domain coverage; ii) we have employed a powerful semi-supervised learning technique to identify high confidence negatives, which increase the model quality; and iii) our approach is based on a non-linear model to address the issue of the correlation between amino acid positions.

### Genome-wide prediction of PDZ domains

We performed a genome-wide prediction of PDZ domain mediated interactions in human and mouse proteomes. The idea was to identify novel interaction partners for human and mouse PDZ domains. In order to do so, we extracted the set of peptides from UniProtKB/Swiss-Prot database [31], release 2013-01, which is a manually curated and reviewed database. We retrieved 20248 and 16597 proteins from human and mouse proteomes, respectively. The last 5 C-terminal residues were taken from each protein to build the peptide sets separately for human and mouse. In this analysis we have used prior knowledge to avoid peptides that are not likely to interact with their respective PDZ domains. Therefore, we considered two filters for selecting the probable binding peptides for a given PDZ domain: i) structural location of the peptides and ii) co-cellular localization of domain and peptide containing proteins.

Previous study showed that PDZ domains have a tendency to bind with intrinsically unstructured proteins (IUPs) [42], thus we considered only those peptides that reside in a disordered segment of a protein. For determining the structural disorder of a protein region we ran the *IUPred* algorithm over the full-length protein sequence to get a disorder score between 0 and 1 for each residue of a protein [43]. Finally, an average score for the last 5 residues (peptide sequence in our study) was obtained to determine putative candidate regions for interaction. A cutoff value of 0.4 was chosen based on the analysis done by *Akiva et al*. [42]. To this end, we ignored all the peptides having the *IUPred* score less than 0.4.

As a second filter, co-cellular localization was applied to avoid unlikely interactions. More clearly, we have considered only those interactions where the peptide containing proteins and the PDZ containing proteins share at least one cellular localization term annotated in Gene Ontology Database [44].

Finally, the eligible peptides were scored by the trained models and sorted according to SVM scores. We have observed C-terminal peptide (IETHV) from Connector enhancer of kinase suppressor of ras 2 protein (Q8WXI2-Human, Q80YA9-Mouse) was targeted by 40 PDZ domains, represented 16 families, in human and mouse. See Additional file 1: Table S3 and Table S4 for top five peptides targeted by most number of human and mouse PDZ domains. The top predictions for each human and mouse PDZ domains are freely available to the scientific community.

## Conclusions

In our comprehensive study, we propose a cluster based computational method to accurately predict the binding partners of PDZ domains using support vector machine (SVM). First, we used an efficient MCL algorithm to cluster all PDZ domains from different organisms and thereafter built prediction models for each PDZ family found by our clustering. Our method offers the largest number of prediction models for PDZ domains to date. We showed that our clustering method maximizes the training datasets, which is important to build powerful prediction models. In the clustering method, we combined all the PDZ domains that share high sequence identity and therefore have similar binding specificity. There are, however, additional cases where the binding preference is very similar despite a low sequence identity. For example, MAGI1-5 and MAGI3-4 domains share similar specificity despite of low sequence identity (24% in mouse) [27]. Since these cases are hard to detect automatically, we used a conservative approach by considering a threshold of 50% sequence identity. Even using this conservative threshold, we were able to achieve a very good prediction accuracy. We also applied semi supervised learning (SSL) strategy for selecting high confidence negative data to re-balance our training sets. In our study, we have developed models based on two feature encoding methods; i) sequence-based and ii) contact-based methods. Since the sequence-based approach does not depend on domain-peptide complex structures, it covers more PDZ domains but may fail to predict binding peptides of a mutated domain with completely different specificity. For mutated domains we can efficiently use our contact-based approach, which considers binding pairs of domain and peptide and thus should be able to more precisely evaluate the effect of mutations. Our method is also able to predict binding peptides of newly characterized PDZ domains. Moreover, our approach is general enough to be easily applicable to other PRMs (i.e. SH2, SH3, WW etc.). We compared our tool with published state-of-the-art methods and achieved better performance. Finally, we performed a genome-wide analysis and predict several novel interactions for human and mouse PDZ domains. The predictive models and the genome-wide top predictions are freely available to scientific researchers.

## Availability and requirements

Models are available under the URL:

http://www.bioinf.uni-freiburg.de/Software/PDZPepInt/PDZPepInt.tar.gz

Genome-wide predictions are available under the URL:

http://www.bioinf.uni-freiburg.de/Software/PDZPepInt/Genome-wide-predictions.tar.gz

Datasets are available under the URL:

http://www.bioinf.uni-freiburg.de/Software/PDZPepInt/PDZ-datasets.tar.gz

Operating systems: Linux

## Electronic supplementary material

Additional file 1: Figure S1: Peptide logos for each PDZ domain cluster. Figure S2: Performance comparison of sequence-based and contact-based approach. Table S1: Performance of sequence-based approach. Table S2: Performance of contact-based approach. Table S3: List of proteins that targeted by highest number of PDZ domains in human. Table S4: List of proteins that targeted by highest number of PDZ domains in mouse. (PDF 336 KB)
