# Supplementary material for: Cluster based prediction of PDZ-peptide interactions
Source: BMC Genomics. 2014 Jan 24;15(Suppl 1):S5. doi: 10.1186/1471-2164-15-S1-S5 (PMC4046824; doi:10.1186/1471-2164-15-S1-S5)
Supplement: Supplementary file 1 — Additional file 1: Figure S1: Peptide logos for each PDZ domain cluster. Figure S2: Performance comparison of sequence-based and contact-based approach. Table S1: Performance of sequence-based approach. Table S2: Performance of contact-based approach. Table S3: List of proteins that targeted by highest number of PDZ domains in human. Table S4: List of proteins that targeted by highest number of PDZ domains in mouse. (PDF 336 KB) [file 12864_2014_5678_MOESM1_ESM.pdf]

Additional Information

Figure S1: Peptide logos for each PDZ domain family. WebLogo [1] was used for constructing the peptide logos. Different families show different ligand binding specificity. Families with at least 10 positive interactions were used for sequence logo.

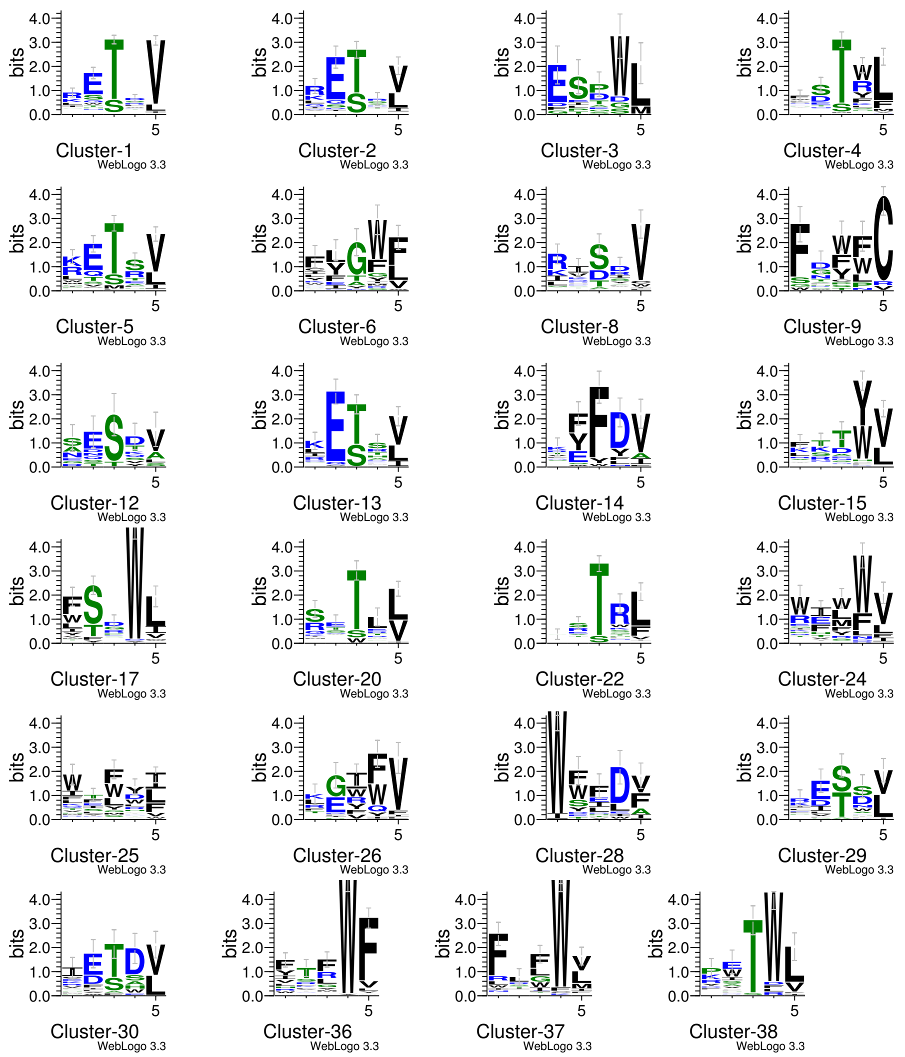

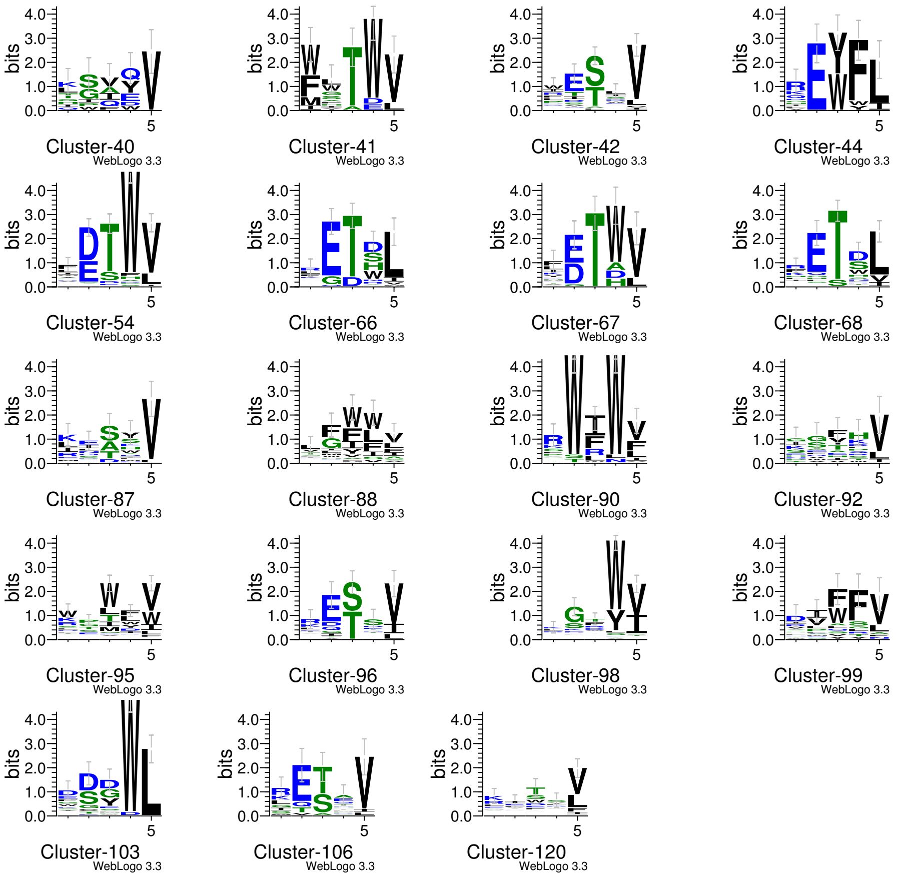
Cluster-40
Cluster-41
Cluster-42
Cluster-44
Cluster-54
Cluster-66
Cluster-67
Cluster-68
Cluster-87
Cluster-88
Cluster-90
Cluster-92
Cluster-95
Cluster-96
Cluster-98
Cluster-99
Cluster-103
Cluster-106
Cluster-120
WebLogo 3.3
bits

Figure S2: (A) The AUC-ROC and (B) the AUC-PR curve obtained by sequence-based feature encoding (red line) and contact-based feature encoding (green dashed line) method.

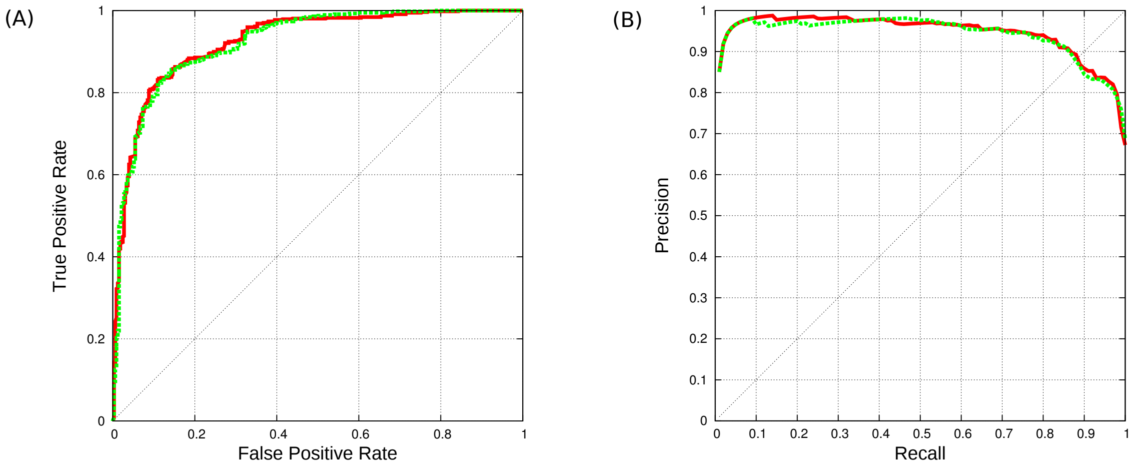

Table S1: Predictive performances of sequence-based approach.

PDZ cluster	Positive int.	Sensitivity	Specificity	Precision	AUC PR	AUC ROC
1	147	0.94	0.72	0.93	0.97	0.92
2	37	0.77	0.89	0.91	0.95	0.94
4	85	0.91	0.83	0.97	0.98	0.87
5	54	0.69	0.75	0.78	0.85	0.83
6	27	0.75	1	1	0.97	0.95
8	36	0.55	0.93	0.9	0.87	0.87
13	27	0.73	0.95	0.93	0.96	0.96
15	51	0.81	0.93	0.95	0.95	0.95
20	67	0.92	0.71	0.93	0.97	0.91
22	67	0.81	0.94	0.95	0.97	0.95
29	29	0.8	0.65	0.76	0.87	0.78
30	13	0.33	0.9	0.27	0.79	0.83
37	60	0.97	1	1	1	0.99
41	12	0.75	1	1	0.98	0.99
42	37	0.42	0.94	0.79	0.79	0.85
54	53	0.87	0.97	0.97	0.99	0.98
66	36	0.98	0.96	0.98	1	1
68	50	0.96	0.63	0.92	0.98	0.91
87	19	0.62	0.71	0.52	0.82	0.82
96	31	0.9	0.76	0.9	0.89	0.8
98	81	0.9	0.94	0.97	0.98	0.95
120	55	0.78	0.57	0.86	0.88	0.73

Table S2: Predictive performances of contact-based approach.

PDZ cluster	Sensitivity	Specificity	Precision	AUC PR	AUC ROC
1	0.94	0.79	0.95	0.97	0.92
2	0.76	0.88	0.88	0.95	0.94
20	0.92	0.68	0.92	0.98	0.94
42	0.53	0.9	0.78	0.73	0.79
54	0.88	0.98	0.98	0.99	0.99
120	0.84	0.44	0.84	0.9	0.77

Table S3: Predicted binding peptides targeted by the highest number of PDZ domain in human.

UniProt-ID	Peptide	targeted by number of PDZ domains
Q8WXI2	`IETHV`	40
Q14524	`RESIV`	39
Q14957	`LESEV`	38
Q9NYB5	`KETQL`	34
P35354	`RSTEL`	34

Table S4: Predicted binding peptides targeted by the highest number of PDZ domain in mouse.

UniProt-ID	Peptide	targeted by number of PDZ domains
Q9ERB5	`KETRL`	42
Q80YA9	`IETHV`	40
O08911	`KETAL`	39
Q01098	`LESEV`	38
Q9JJV9	`RESIV`	37

References

[1] Gavin E. Crooks, Gary Hon, John-Marc Chandonia, and Steven E. Brenner. WebLogo: a sequence logo generator. *Genome Res*, 14(6):1188–90, 2004.
